# A Refractory, Infected Lung Bulla and an Abscess Treated Using Percutaneous Drainage in a Patient With Human T-Lymphotropic Virus Type 1-Associated Myelopathy

**DOI:** 10.7759/cureus.20333

**Published:** 2021-12-10

**Authors:** Kaho Sugahara, Toyoshi Yanagihara, Yuri Nakamura, Yuuka Nakayama, Katsuzo Hanaoka, Migiwa Ohgushi, Naruhiko Ogo, Yu Inutsuka, Tatsuma Asoh, Yukiko Harada, Reiko Yoneda, Takashige Maeyama

**Affiliations:** 1 Respiratory Medicine, Hamanomachi Hospital, Fukuoka, JPN; 2 Neurology, Brain and Nerve Center, Fukuoka Central Hospital, Fukuoka, JPN; 3 General Medicine, Hamanomachi Hospital, Fukuoka, JPN; 4 Pathology, Hamanomachi Hospital, Fukuoka, JPN

**Keywords:** aspergillus, pseudomonas aeruginosa, mrsa, htlv-1-associated arthritis, htlv-1-associated myelopathy, percutaneous drainage, infected lung bulla

## Abstract

We present a case of a 54-year-old Japanese woman with established human T-lymphotropic virus type 1 (HTLV-1)-associated myelopathy who developed a refractory infected lung bulla and lung abscess caused by *Pseudomonas aeruginosa*, Methicillin-resistant *Staphylococcus aureus*, and Aspergillusspecies. Since antibiotic treatment alone failed to resolve the infection, percutaneous drainage of the infected bulla was performed. Although a prolonged treatment period was necessary, the infected lung bulla and the lung abscess were eventually resolved. During her illness, the patient also developed arthritis, possibly related to the HTLV-1 infection. Thus, persons infected with HTLV-1 can develop refractory infections, myelopathy, and arthritis. Percutaneous drainage is an option to treat refractory infected lung bullae.

## Introduction

Human T-lymphotropic virus type 1 (HTLV-1), a human retrovirus discovered in 1980, is a causative pathogen of adult T-cell leukemia/lymphoma (ATL) and several HTLV-1-associated organ dysfunctions, including myelopathy, pulmonary diseases, and arthritis. HTLV-I is primarily transmitted by breastfeeding, although can spread via blood transfusion, sharing of needles, and sexual intercourse. HTLV-1 is distributed globally and is estimated to infect five to 10 million people worldwide [1]. The virus is highly prevalent in some regions, including southern Japan, the Caribbean, South America, the Melanesian islands, the Middle East, and Africa [2]. In these areas, prevalence ranges from 3% to 5% in Trinidad, and up to 30% in rural areas in Kyushu island in Japan [3]. In contrast, the United States and Europe have low prevalence rates of less than 1% [4].

HTLV-1-associated myelopathy (HAM) is characterized by progressive spastic weakness of the lower limbs, neurogenic bladder, and lower back pain, thus imposing a substantial health burden [5]. The lifetime risk of developing HAM has been reported as 0.25% or higher in HTLV-1-infected individuals, based on ethnic differences [6-8]. Treatment of HAM remains unsatisfactory. Although potential disease-modifying drugs such as corticosteroids are widely used, their clinical efficacy is unproven. Not much is known about the precise natural courses of patients with HAM. Here, we report a case of a refractory infected lung bulla and lung abscess that required percutaneous drainage in addition to long-term antibiotics in a patient with HAM. This case report is helpful to understand the complicated clinical course of an HTLV-1-affected patient.

## Case presentation

A 54-year-old Japanese woman was transferred to Hamanomachi Hospital, Fukuoka, Japan, for persistent fever with chest imaging abnormalities. She had a smoking history of 30 pack years. She had been diagnosed with oropharyngeal cancer and had been treated with chemoradiotherapy five years earlier. Two years prior to this clinical presentation, the patient had an established diagnosis of HTLV-1-associated myelopathy (HAM), with neurological findings of neurogenic bladder, orthostatic hypotension, bilateral lower limb spasticity, increased deep tendon reflexes, and positive bilateral Babinski reflexes, as well as positive serum and cerebrospinal fluid tests for anti-HTLV-1 antibodies.

Post diagnosis of HAM, the patient experienced recurrent episodes of aspiration pneumonia and had a gastrostomy placed a year and a half earlier. A year earlier, she developed acute progressive HTLV-1-related myelopathy of bilateral lower limbs and was treated with methylprednisolone 1000mg pulse therapy, followed by 5mg of prednisone maintenance therapy at Fukuoka Central Hospital, Fukuoka, Japan. In the outpatient clinic at Fukuoka Central Hospital, she presented a fever with mild sputum. Her chest computed tomography (CT) showed infiltration in the upper right lobe and she was diagnosed with pneumonia. Despite treatment with levofloxacin for five days, a high fever persisted, and she was admitted to Fukuoka Central Hospital.

**Figure 1:**
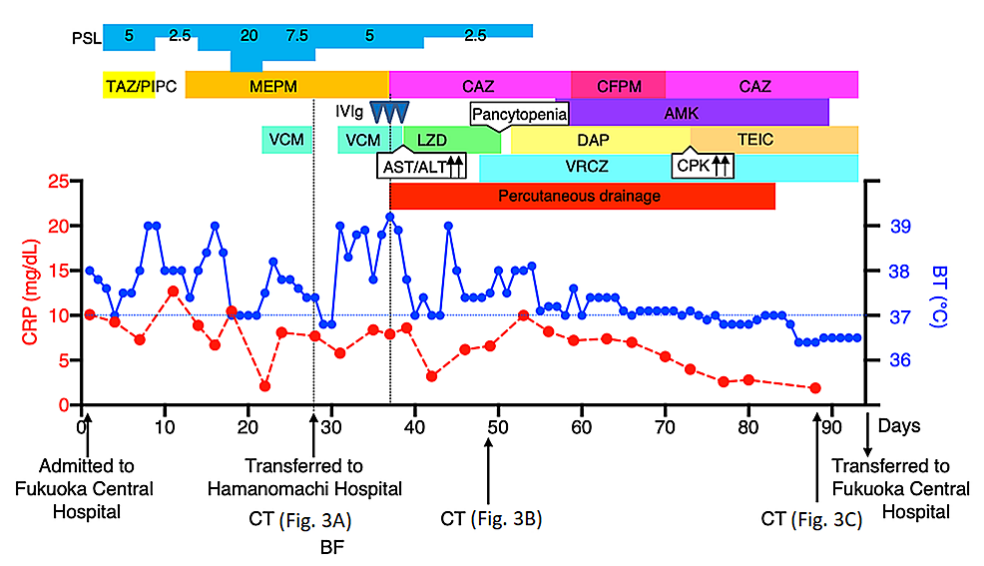
Clinical course of the patient. The patient was transferred to Hamanomachi Hospital on day 28. PSL: prednisolone; IVIg: intravenous immunoglobulin; TAZ/PIPC: tazobactam/piperacillin; MEPM: meropenem; CAZ: ceftazidime; CFPM: cefepime; AMK: amikacin; VCM: vancomycin; LZD: linezolid; DAP: daptomycin; TEIC: teicoplanin; VRCZ: voriconazole; CRP: C-reactive protein; BT: body temperature; BF: bronchoscopy

At Fukuoka Central Hospital, she was placed on total parenteral nutrition, suspected of aspiration pneumonia, and was treated with tazobactam/piperacillin (TAZ/PIPC), followed by meropenem (MEPM) and vancomycin (VCM). She was also suspected to have vasculitis from the findings of purpura on her both lower limbs, and the corticosteroid dose was temporarily increased. Despite this effort of examinations and treatment for one month, her fever, high levels of serum C-reactive protein (CRP), and chest imaging abnormalities persisted. She was then transferred to Hamanomachi Hospital for further investigation and treatment on hospital day 28 (Figure 1).

**Figure 2:**
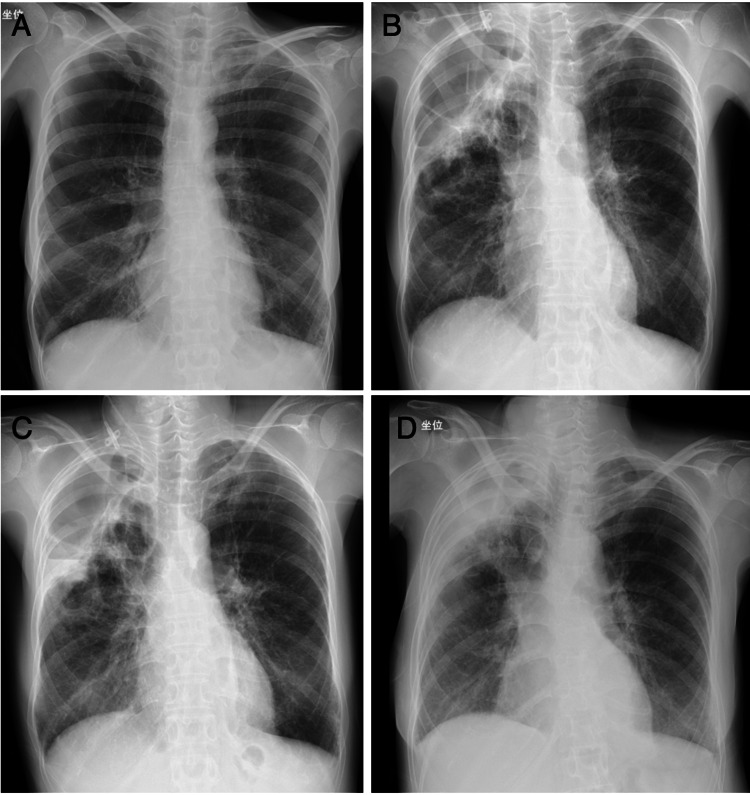
Chest x-ray images of the patient. Chest x-ray images (A) one year earlier, (B) on admission to Hamanomachi Hospital, (C) before percutaneous drainage on day 38, and (D) before discharge from Hamanomachi Hospital on day 87.

**Figure 3:**
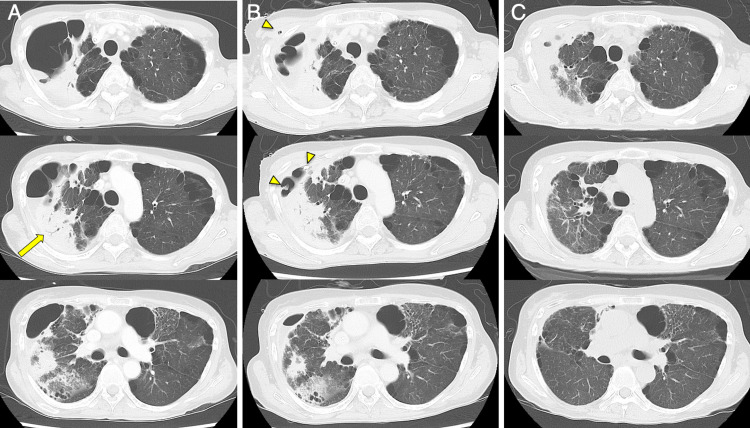
Chest CT images of the patient. Chest CT images (A) on admission to Hamanomachi Hospital, (B) during percutaneous drainage on day 50, (C) before discharge from Hamanomachi Hospital on day 88. An arrow indicates the lesion where trans-bronchial lung biopsy was performed. Arrowheads indicate a percutaneous drainage tube inserted into the lung bulla.

On admission to Hamanomachi Hospital, she was sarcopenic with a body mass index of 13.5. She had muscle weakness of manual muscle testing (MMT) of 2-3/5, mainly in her lower limbs, which were the same levels of her baseline. Her respiratory condition was normal, with a respiratory rate of 15/min and SpO_2_ 98% on room air. Crackles were not detected in her chest. Persistent, increased levels of CRP (7.7 mg/dL) and a white blood cell count of 6400 /μL were noted (Figure 1). Chest x-ray imaging showed a giant air-space in the right upper lung field one year earlier (Figure 2, panel A). Consolidation was found in the right upper lung field on admission to Hamanomachi Hospital (Figure 2, panel B). Chest CT imaging showed dense consolidation with an air bronchogram and a giant bulla with fluid retention in the right upper lobe, as well as an emphysematous change in the bilateral lungs (Figure 3, panel A). Given the clinical course at Fukuoka Central Hospital and examination results on admission to Hamanomachi Hospital, a refractory lung abscess and an infected bulla were suspected. Suspected pathogens were *Pseudomonas aeruginosa* and Methicillin-resistant *Staphylococcus aureus* (MRSA), which were detected by sputum culture at Fukuoka Central Hospital. The differential diagnoses were opportunistic infections such as aspergillosis, mycobacteriosis, vasculitis, HTLV-1-associated bronchioalveolitis, and adult T-cell leukemia infiltration.

**Figure 4:**
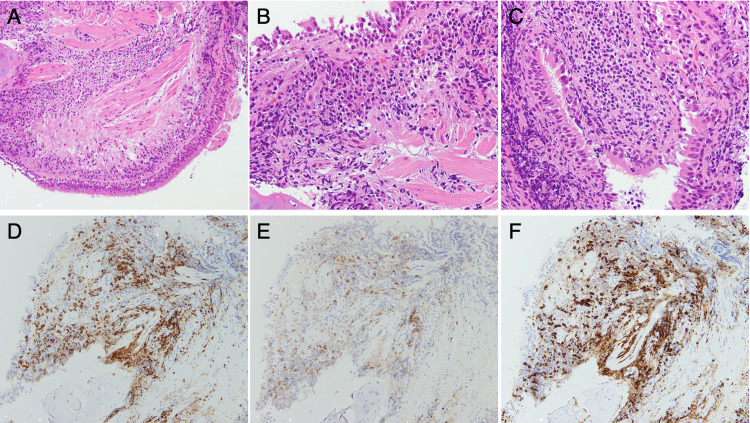
Pathological examination. (A-C) Hematoxylin and eosin (HE) staining. Bronchial tissue with mild-to-moderate chronic inflammation was collected. Infiltrating lymphocytes were small. Some neutrophils and plasma cells were also seen. No findings suggestive of malignancy or adult T-cell lymphoma were noted. Immunohistochemistry staining for (D) CD3, (E) CD4, (F) CD8. Magnification: (A, D-F) ×200, (B and C) ×400. CD: cluster of differentiation

To confirm the diagnosis, a bronchoscopy was performed on hospital day 30 (Figure 1). Transbronchial lung biopsies (TBLB), bronchial brushing, and bronchial washing were conducted from the right B2 bronchus. Bacterial culture from bronchial washing was positive for *Pseudomonas aeruginosa* and MRSA, consistent with the sputum culture at Fukuoka Central Hospital and on admission to Hamanomachi Hospital. Neither Aspergillus species nor Mycobacterium species was detected. Hematoxylin and eosin staining of TBLB showed bronchial tissues with mild to moderate chronic inflammation and lymphocyte-predominant infiltration (Figure 4, panels A-C). Infiltrating lymphocytes were small and showed no morphological abnormalities. A small number of neutrophils and plasma cells were also seen. No findings suggestive of malignancy or adult T-cell lymphoma were noted. Infiltrating lymphocytes were a mixture of cluster of differentiation (CD)20-positive B lymphocytes and CD3-positive T lymphocytes (Figure 4, panel D), with a slight predominance of B lymphocytes. CD8-positive T lymphocytes were predominant among T lymphocytes (Figure 4, panels E and F). The pathological meaning is explained in the Discussion section.

**Figure 5:**
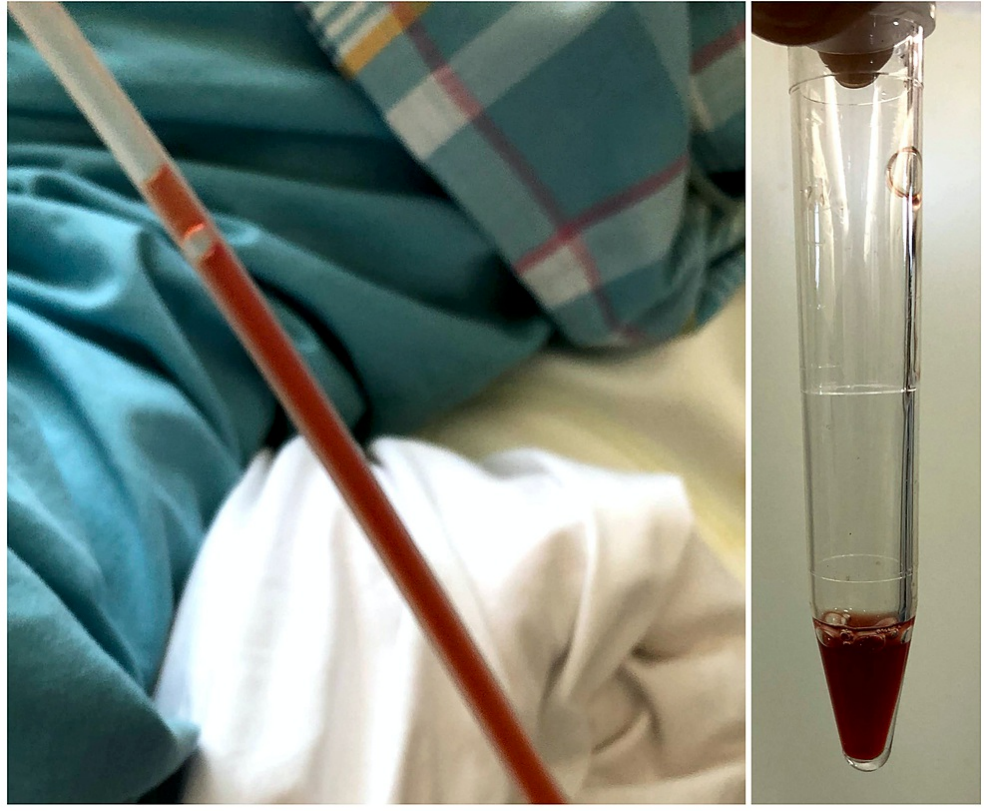
Appearance of the drainage fluid. Drainage fluid from the percutaneous drain in the infected bulla in the right upper lobe of the lung was reddish-brown and cloudy.

Based on these results, the patient was initiated with antibiotics (MEPM and VCM, followed by ceftazidime and linezolid). She was also treated with 5 g/day of intravenous immunoglobulin for three days, considering her immunosuppressive condition. However, she remained febrile with the upward trend of CRP. Considering the refractory state of the infected lung bulla, we performed percutaneous ultrasound-guided drainage of the bulla on day 38, following daily washing using sterile saline (Figure 2, panel C and Figure 3, panel B). The fluid from the drain was reddish-brown and cloudy, and *Aspergillus* species were detected in the drainage culture (Figure 5). Given the pulmonary aspergillosis in addition to *Pseudomonas aeruginosa* and MRSA infection, voriconazole was initiated on day 49, although beta-D-glucan (14.3 pg/mL) and galactomannan (0.1 index) were negative.

During treatment, we had to change antibiotics several times because of the drug resistance of *Pseudomonas aeruginosa* (carbapenem and quinolone resistance with moderate cephem resistance) and the side effects of the antibiotics. Amikacin was used for the combination treatment of drug-resistant *Pseudomonas aeruginosa* from day 60. Complications arising from anti-MRSA drugs included serum AST/ALT elevation caused by VCM, pancytopenia triggered by linezolid, and serum creatine kinase (CK) elevation (>1000 U/L) in response to daptomycin (Figure 1).

Prednisolone was gradually reduced and discontinued in consideration of its adverse effect on refractory infections. On approximately day 70, she gradually developed polyarthritis in her knees, right shoulder, and both wrist joints. Serological examination revealed elevation of rheumatoid factor (59.7 IU/L), but no elevation of anti-CCP antibodies (0.8 U/mL). Joint ultrasound showed active synovitis in both hand joints. The differential diagnosis of her poly-arthritis was antibiotic use-related arthritis, sepsis-related arthritis, pseudogout crystal arthropathy, rheumatoid arthritis, and HTLV-1-associated arthritis. She was treated with non-steroidal anti-inflammatory drugs (NSAIDs). Corticosteroids or other immunomodulating drugs could not be used until the infected bulla and lung abscess were completely healed. Her poly-arthritis persisted several months after the transfer to Fukuoka Central Hospital. From these results and clinical course, she was considered to have rheumatoid arthritis or HTLV-1-associated arthritis, which had been suppressed by the corticosteroid treatment.

Thereafter, the fever gradually resolved, and the inflammatory response trended downwards. Chest imaging showed improvement of infiltration and reduction of the size of the infected bulla (Figure 2, panel D and Figure 3, panel C). We removed the drain tube on day 87. Her general condition improved, and she was transferred to Fukuoka Central Hospital on day 94. She was discharged to her home on day 150. No relapse of the infection has been observed since the discontinuation of antibiotics.

## Discussion

We describe an HTLV-1-affected patient who developed several complications. The patient needed to be transferred to Hamanomachi Hospital for further investigation of uncertain lung abnormalities with inflammation, since there were several other differential diagnoses, such as mycobacteriosis, ANCA-associated vasculitis, HTLV-1-associated bronchioalveolitis, and adult T-cell leukemia infiltration [9]. We were then able to make a final diagnosis of an infected lung bulla and a lung abscess, using bronchoscopy and percutaneous drainage.

The patient had numerous lung bulla. Her smoking history might be the main reason for the bullous change. Further, HTLV-1-infected status might also have caused developing such a giant bulla, since HTLV-1 associated bronchioloalveolar disorder often indicates progression of air trapping [10].

Regarding lung pathology, we observed lymphocyte-predominant inflammation from lung tissues in the right upper lobe. Typically, biopsies of lung abscesses show neutrophilic granulocytes with dilated blood vessels and inflammatory edema in the acute phase [11]. In the chronic phase, lymphocytes, plasma cells, and histiocytes are present around a layer of pyogenic membrane surrounding the abscess cavity, which is filled with pus [11]. In this case, lung biopsies from the patient could have been pathological findings of the chronic lung abscess. However, the patient’s status as an HTLV-1 carrier might have affected the findings. Given that HTLV-1 primarily infects CD4+ T-cells and is thought to alter their functions and lineages, some infiltrated T-cells might have been dysfunctional, partially explaining the patient’s refractory infection. Other possible reasons included (a) an immunosuppressive effect of corticosteroid treatment, (b) HAM and (c) sarcopenia that caused muscle weakness, resulting in decreased ability to expel sputum, and (d) oropharyngeal dysfunction after treatment for oropharyngeal cancer, which might have resulted in repeated asymptomatic aspiration.

We performed percutaneous catheter drainage of the infected bulla. There were concerns regarding potential complications, e.g., pneumothorax or bleeding, of percutaneous drainage for lung parenchyma, which differs from drainage for pyothorax. However, 10 days after transfer to Hamanomachi Hospital, we determined that the infection could not be improved without drainage. Several reports have documented the utility of percutaneous drainage of infected bullae [12-14]. Further, we thought that the visceral pleura adjacent to the infected bulla might be adhering to the parietal pleura due to inflammation, which would have prevented pneumothorax after percutaneous drainage for the bullae. Based on these previous reports and findings, we decided to perform percutaneous drainage. As a result, we were able to discover evidence of Aspergillus co-infection and were able to manage the refractory infection. Percutaneous drainage can thus be an option to treat refractory infected lung bullae.

During admission, the patient developed arthritis of multiple joints, which was later considered to be rheumatoid arthritis or HTLV-1-associated arthritis. ﻿The prevalence of HTLV-1 infection is higher in patients with rheumatoid arthritis than in healthy controls [15]. Chronic rheumatic diseases, including arthritis, occur in transgenic mice with the HTLV-1 genes, Tax, and HBZ [16,17]. Importantly, attenuated effectiveness of tumor necrosis factor inhibitors for HTLV-1-positive patients with rheumatoid arthritis has been reported [18]. ﻿HTLV-1 infection thus affects the clinical course of patients who develop arthritis.

## Conclusions

HTLV-1 is a causative pathogen of ATL and several HTLV-1-associated organ dysfunctions, including HAM, bronchioalveolitis, and arthritis. The case highlights that these HTLV-1-associated organ dysfunctions can be complicated in one patient, although no case reports have been published. The case also highlights the importance of the infectious disease principle of "source control," that is, percutaneous drainage for the treatment of refractory infected lung bullae.
